# Modulation of Function, Structure and Clustering of K^+^ Channels by Lipids: Lessons Learnt from KcsA

**DOI:** 10.3390/ijms21072554

**Published:** 2020-04-07

**Authors:** María Lourdes Renart, Ana Marcela Giudici, Clara Díaz-García, María Luisa Molina, Andrés Morales, José M. González-Ros, José Antonio Poveda

**Affiliations:** 1 Instituto de Investigación, Desarrollo e Innovación en Biotecnología Sanitaria de Elche (IDiBE), and Instituto de Biología Molecular y Celular (IBMC), Universidad Miguel Hernández, Elche, E-03202 Alicante, Spain; 2 iBB-Institute for Bioengineering and Bioscience, Instituto Superior Técnico, Universidade de Lisboa, 1049-001 Lisboa, Portugal; 3 Departamento de Fisiología, Genética y Microbiología, Universidad de Alicante, E-03080 Alicante, Spain

**Keywords:** lipid–protein interactions, C-type inactivation, membrane protein folding, ion channel clustering, ion binding, KcsA modulation

## Abstract

KcsA, a prokaryote tetrameric potassium channel, was the first ion channel ever to be structurally solved at high resolution. This, along with the ease of its expression and purification, made KcsA an experimental system of choice to study structure–function relationships in ion channels. In fact, much of our current understanding on how the different channel families operate arises from earlier KcsA information. Being an integral membrane protein, KcsA is also an excellent model to study how lipid–protein and protein–protein interactions within membranes, modulate its activity and structure. In regard to the later, a variety of equilibrium and non-equilibrium methods have been used in a truly multidisciplinary effort to study the effects of lipids on the KcsA channel. Remarkably, both experimental and “in silico” data point to the relevance of specific lipid binding to two key arginine residues. These residues are at non-annular lipid binding sites on the protein and act as a common element to trigger many of the lipid effects on this channel. Thus, processes as different as the inactivation of channel currents or the assembly of clusters from individual KcsA channels, depend upon such lipid binding.

## 1. Introduction

Ion channels are a superfamily of membrane proteins involved in a myriad of cellular processes such as the control of cell excitability, signal transduction, muscle contraction or synaptic transmission. Thus, their dysfunction is associated with numerous diseases related to the nervous, cardiovascular, respiratory, endocrine, urinary and immune systems, and has been termed channelopathies [1,2,3].

**Figure 1:**
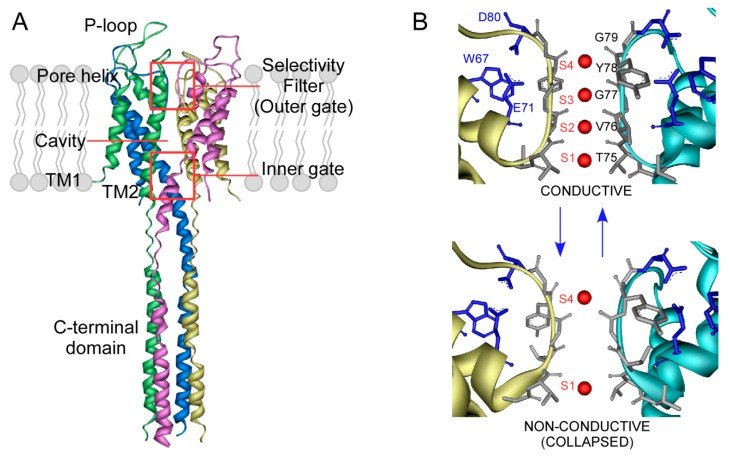
Scheme of the KcsA structure. (**A**) Crystallographic structure of the full-length KcsA in the closed state (PDB entry: 3EFF). Main structures and domains are highlighted. (**B**) Zoom of the selectivity filter (SF) structure, including the signature sequence TVGYG. The inactivation triad (E71-D80-W67) location is also shown in blue sticks. The SF can adopt a non-conductive or collapsed conformation at low K^+^ concentration (<5 mM) (PDB entry; 1K4D). Increasing the amount of the permeant cation leads to a shift of the equilibrium to a conductive state, where all four K^+^ binding sites can be identified (PDB: 1K4C).

Our knowledge on these proteins has increased exponentially in the last decades since the atomic structure of a few ion channels has been solved, mainly through X-ray crystallography and nuclear magnetic resonance (NMR) spectroscopy. KcsA, a potassium channel from the *Streptomyces lividans* bacteria, was the first ion channel whose atomic structure was solved [4,5,6]. This fact, the relative ease to purify it in relative large amounts, and its endurance to experimental conditions have made KcsA the main model to study the structure/function relationships in ion channels. This protein is formed by four identical subunits arranged around a central aqueous pore through which ions flow. Each subunit comprises cytosolic N-terminal and C-terminal domains and two α-helical transmembrane segments (TM1 and TM2). Between the two transmembrane segments there is a short, tilted helix spanning inward to about one-third of the membrane thickness and oriented towards the centre of the tetramer, followed by a stretch of amino acids containing the consensus sequence _75_TVGYG_79_, known as the selectivity filter (SF). This is the narrowest part of the protein pore and, as its name reveals, is highly selective to define which kind of ions can pass through it. Thus, it allows K^+^ permeation, while preventing the physiologically relevant Na^+^ from flowing through the pore. Below it, an aqueous vestibule forms, shaped by the TM2 segments. There, ions are fully hydrated and become partly stabilized near the inner mouth of the narrower selectivity filter by the four dipolar pore helices, which point their negatively charged C-terminal ends toward the hydrated ions (Figure 1A). However, residues lining this cavity exhibit a low affinity by surrounding ions and only modestly discriminate between Na^+^ and K^+^.

In the resting state, the TM2 segments form a cross-section close to their C-terminal through electrostatic interactions, which effectively prevent passage of ions. However, a low intracellular pH causes the protonation of residues involved in such interactions, triggering a large hinge-bending motion away from the fourfold symmetry axis that opens the aqueous vestibule to the cytoplasmic media, eventually allowing ion flow. This open channel state, however, is not stable and the selectivity filter evolves within a few seconds from a resting conductive state to a non-conductive conformation known as the inactivated state, thus determining the time during which the channel is active. Three residues, E71, D80 and W67, called the inactivation triad, have been identified as key elements to stabilize this conformation [7,8,9]. They are located just behind the selectivity filter and interact through a web of hydrogen bonds between themselves and the selectivity filter, thus, influencing its conformation (Figure 1B). Once the pH is restored to neutrality, TM2 segments cross back to the closed position while the selectivity filter again adopts the resting conductive state. Thus the concerted movement of these two gates, the TM2 crossing (inner gate) and the selectivity filter (outer gate), defines the functional cycle of KcsA [10].

One intriguing question about potassium channels is related to their capability to conduct K^+^ at very high rates, near the diffusion limit, while maintaining a very high selectivity for K^+^ versus Na^+^. Another question requiring further definition has to do with the conformation of the SF and other channel domains when in the inactivated state. The selectivity filter is a key element in both processes and thus, many efforts have been made to define its conformational plasticity in the presence of different concentrations of conductive and non-conductive ions. From the experimental viewpoint, X-ray studies on detergent-solubilized KcsA show that the selectivity filter adopts two different conformational states: conductive and non-conductive or collapsed, which can switch over depending on K^+^ concentration [4]. The conductive state appears at high potassium concentration, showing four K^+^ binding sites termed S1–S4, from the inner to the outer side of the filter (Figure 1B). In this state, the average occupation of the conductive filter is of two alternating K^+^, either in the configuration S1-S3 or S2-S4, where water molecules apparently occupy the other two positions, enabling K^+^ flow. Additionally, the permeation process has also been simulated by molecular dynamics (MD) techniques [11,12,13]. These studies have provided new additional details, particularly on the dynamics of cations within the filter, and have raised new issues, still under debate, on whether intercalated water molecules accompany the cations through the filter during permeation. Conversely, X-ray data at a low potassium concentration (<5 mM), detects a collapsed filter conformation. The α-carbon of G77 is now twisted inward, narrowing the central part of the filter and eventually eliminating the S2 and S3 sites, thus, impeding ion flow. In this collapsed state an average of only one K^+^ is found at either S1 or S4 sites. This non-conductive conformation has been associated with the inactivated state [14,15,16]. However, there is still controversy on this matter, as other authors point out to an inactivated state, which is not collapsed but somewhat similar to the resting-conductive state [17,18,19]. In the presence of the non-permeant Na^+^, the filter adopts a similar collapsed conformation, with an average occupation of one Na^+^ bound either to S1 or S4 sites, but being unable to induce the concentration-dependant transition to the conductive conformation [20]. Rb^+^ and Cs^+^ are also permeant cations and, therefore, able to induce the transition to the conductive conformation at high concentrations, but they are unable to occupy the S2 site [21]. This particular feature has been related to their lower current amplitude and slower inactivating rate. The rationale being that these slow-paced ions spend a longer time inside the selectivity filter due to the higher energy needed to leap over the S2 site [5,22,23].

Many integral membrane proteins are modulated structurally and/or functionally by the surrounding lipids within the membrane. However, there are only a few cases where the molecular mechanism through which this modulation occurs has been deciphered [24]. Actually, KcsA is one of the best studied ion channels undergoing lipid modulation. Along this review, we will depict different approaches used to study this sort of modulation, including NMR, fluorescence, electrophysiology or molecular dynamics. A special emphasis is placed on the use of a thermal denaturation fluorescence assay, since the variation of the experimental observable is very large and covers a broad range of ligand concentration. From all these studies it can be concluded that lipids are able to alter different KcsA features, although interestingly most of such alterations have in common the initial binding of anionic lipids to two key arginine residues located at non-annular lipid binding sites on the channel protein (see below). On one hand, such anionic lipid binding influences the interaction of the selectivity filter with permeant and non-permeant cations to finally modify the channel inactivation pattern. On the other hand, bound anionic lipids alter protein assembly processes, such as the association/dissociation of functionally distinct channel clusters from individual KcsA channels through lipid-competing, channel–channel interactions.

## 2. Lipids as Modulators of KcsA Function

**Figure 2:**
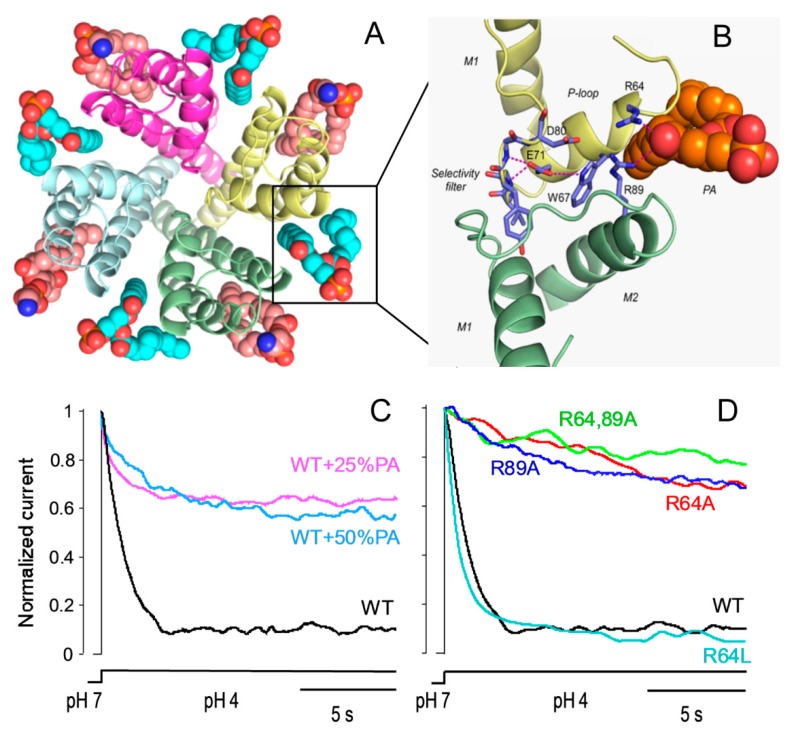
Lipid modulation of KcsA inactivation by binding of non-annular anionic lipids. In (**A**), a top view molecular model of KcsA with annular (acyl chains in light pink) and non-annular (acyl chains in cyan) lipids is shown. In (**B**), a zoom of a non-annular lipid binding site is depicted, with a PA molecule bound. In (**C**,**D**), normalized KcsA macroscopic currents elicited by pH jumps (pH 7 to 4) are shown. (**C**) The influence of anionic phospholipid on KcsA inactivation. K^+^ currents were recorded from macropatches of WT KcsA reconstituted in plain asolectin lipids (WT), asolectin lipids with an added 25% of egg PA (WT + 25% PA) or asolectin lipids with an added 50% of egg PA (WT + 50% PA). (**D**) Effects of R64A, R64L, R89A and R64,89A KcsA mutations on channel inactivation upon reconstitution in plain asolectin lipids. The holding potential was set to +150 mV in all these experiments.

As in other membrane proteins, lipids have been identified as modulators of ion channels. Such influence is exerted either through an indirect effect on the protein by altering physical properties of the membrane such as tension, curvature, lateral pressure or the thickness of the lipid bilayer [25,26,27]; or through a direct effect caused by lipid binding to specific sites of the channel [24,28]. In KcsA, anionic phospholipids have been found fundamental to preserve its channel activity [29,30], to raise its chemical and thermal stability, and to achieve proper folding and assembly as a tetrameric protein [31,32,33]. Distinct sites on the channel protein where anionic phospholipids bind have been associated with these effects. For instance, binding to the N-terminal R11 and K14 residues increases the open channel probability and conductance through the stabilization of the inner gate [34]. Likewise, anionic lipid binding to arginines 27, 117, 121 and 122, located at the cytoplasmic side, close to the C-terminal segment, causes an increase in the stability of the channel protein against chemical denaturation [31]. Finally, non-annular lipid binding sites have been found at the interface between adjacent subunits at the extracellular side of the channel (Figure 2). The location and lipid binding features of these non-annular sites, usually at protein clefts and with high specificity, differ greatly from those of the so-called annular lipid sites that would correspond to the transmembrane surface of the protein, in contact with the first layer of the surrounding lipids [35]. There are several examples of lipids bound with sufficiently high affinity to non-annular sites of membrane proteins, as to remain bound upon protein crystallization, being detected in the protein X-ray structure [36,37]. KcsA is one of these cases and a phosphatidylglycerol (PG) molecule was found non-covalently bound to non-annular sites [38] (Figure 2).

Experiments monitoring quenching of the intrinsic fluorescence of KcsA with brominated phospholipids determined a higher affinity of anionic lipids vs. zwitterionic ones to bind these sites and proposed that three out of the four available sites should be occupied for the channel to be active [39]. However, the mechanism through which anionic lipids exert these effects is still a matter of debate. From the crystal structural data, two cationic arginine residues, R64 and R89, flanking the non-annular sites at the extracellular side of the membrane, appear as key elements to bind the anionic lipids. This would explain the higher binding affinity of the non-annular sites for these lipids compared to the zwitterionic ones. Computer simulations have also pointed out that these arginine residues form hydrogen bonds with the hydroxyl, phosphate and carbonyl esters groups of the anionic lipids [32,40]. Based on molecular dynamics simulations, Weinghart and cols. proposed the hypothesis that R64 and R89 could re-orient their sidechains to interact either with the anionic non-annular lipids or with the inactivation triad, thus explaining why these lipids modulate the activity of KcsA [41]. From the kinetic analysis of channel activity at the single-channel level, it has been concluded that phosphatidic acid (PA), an anionic phospholipid, stabilizes the open state of WT KcsA by decreasing the closing rates, thus explaining the slower inactivation rate detected at the macroscopic level. Interestingly, when R64 and/or R89 are mutated to alanine, thus eliminating the cationic charge of the residues, the activity of the channel at either the single-channel or macroscopic level shows a behaviour very similar to that of the WT channel in the presence of PA. However, the R64L mutant, also with a non-charged side chain, but bulkier than alanine, shows an opposite profile. That is, it shows short open times and fast closing rates that end in fast inactivation rates, which is very similar to the WT channel without added PA (Figure 2). In addition, PA has no effect on channel function either at the macroscopic or single-channel level either in the alanine or leucine mutants. These data along with others from MD simulations and NMR from the same samples, strongly support the idea that these residues are the main anchor of anionic lipids to the non-annular sites, and that the modulatory role of these anionic lipids on KcsA mainly occurs through this via [42]. Moreover, these data support the above-mentioned hypothesis, suggesting a pivotal role for these residues. These arginines seem able to either interact with the non-annular anionic lipids, when present at the non-annular sites, or turn towards the channel pore, where they would stabilize the inactivating triad, inducing a faster inactivation. As in the binding of anionic phospholipids, it could be anticipated that the positive charge in the arginine side chain should be essential. However, to stabilize the inactivating triad one key factor is the side-chain length, but not so much the charge, since leucine but not alanine is also able to speed up inactivation. MD simulations of these mutants offer new insights on this matter. According to these data, it is R89 that interacts directly with D80, one element of the inactivating triad, thus stabilizing it and increasing the inactivation rate. When in the presence of anionic lipid, the frequency of this interaction diminishes because R89 would be oriented towards this lipid, H-bonding to its polar headgroup. Consistent with this idea, when the zwitterionic phosphatidylcholine (PC) occupies the non-annular site, its interaction with R64 and R89 becomes highly variable, thus increasing the R89-D80 interaction, which indeed corresponds with the faster inactivation observed experimentally.

## 3. Lipids as Structural Effectors of KcsA

As mentioned in the previous section, lipids can bind to a membrane protein and act as effectors on its conformation and function. Regarding the characterization of the lipid–protein interactions involved, there are few techniques able to quantify (in terms of affinity) this phenomenon. Although electron spin resonance (ESR) is a very useful technique [43,44], fluorescence methods are far more sensitive and simple [45]. In KcsA, the interactions of zwitterionic and anionic phospholipids with the annular transmembrane portions of the protein and non-annular lipid binding sites were first performed by fluorescence quenching methods. Here, the native tryptophan (Trp) residues of the liposome-reconstituted protein were used as fluorescence reporters and different types of brominated lipids as quenchers [46,47]. Those studies revealed that anionic phospholipids can bind to the non-annular sites of the protein with a higher affinity (relative to the PC content in the membrane), and that the binding of at least three of these molecules leads to an increase in the open probability, current amplitude, and mean open times. This is presumably caused by an increase in the probability of the transition from the inactivated to the open state, and/or a decrease in the probability of the opposite, open to inactivated transition [39].

**Figure 3:**
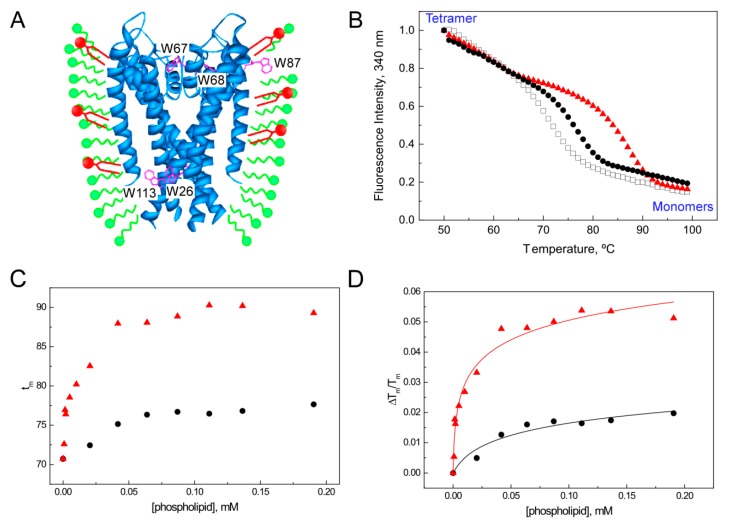
Binding of lipids to KcsA followed by the protein thermal denaturation assay. (**A**) Scheme of the mixed-micelles system, where both lipids (small amounts, in red) and protein are completely solubilized by an excess of detergent (in green). The intrinsic fluorescence from the five Trp residues highlighted in magenta can be used to monitor the thermal denaturation of KcsA. (**B**) Typical thermal denaturation profile of DDM-solubilized KcsA, where the dissociation of the native tetramer follows a sigmoidal behavior. Addition of 0.1 mM of DOPC (black dots) or DOPA (red triangles) results in an increase of the stability of the protein in plain micelles (white squares). (**C**) Dependence of the thermal stability of KcsA WT on phospholipid concentration. Experiments were performed at 0.5 µM protein concentration in 20 mM HEPES (pH 7.0), 1 mM DDM, 100 mM NaCl, increasing the amounts of DOPC or DOPA. (**D**) Solid lines represent the best fits of the experimental data points to the ligand binding model described in Equation (4). The estimated dissociation constants (K_D_) for the single set of binding sites detected in these experiments are 9 ± 6 µM and 0.5 ± 0.2 µM for DOPC and DOPA, respectively.

In order to explore the role of different phospholipids as effectors on the WT KcsA channel structure and stability, our group introduced a thermal denaturation assay performed in a detergent-phospholipid mixed micelles system (e.g., small amounts of lipids solubilized in excess of dodecyl β-D-maltoside (DDM) detergent micelles containing the protein) (Figure 3A). The main advantage of using the mixed-micelle system is to dissect a specific effect of a lipid in a bilayer-free environment, excluding the effects of the membrane properties, such as the thickness and lateral pressure. The thermal denaturation assay monitors the changes in the intrinsic fluorescence of WT KcsA as the temperature increases at a constant rate, which leads to the irreversible dissociation (and partial unfolding) of the tetrameric KcsA into its constituent monomers [32] (Figure 3B). The fluorescence signal results from the average emission of five Trp residues per monomer (see location at Figure 3A). The midpoint temperature of dissociation (*T_m_*) can be determined from the sigmoidal thermal denaturation curves by fitting the data to a two-state unfolding model, assuming a linear dependence of the pre and post transition baselines on temperature:

(1) $$FI_{340}=\frac{\alpha_{N}+\beta_{N\;}\left(T-298\right)+\left(\alpha_{D}+\beta_{D}\;\left(T-298\right)\right)\;e^{\frac{-\Delta H_{D-N}}{R}\;\left(\frac{1}{T}-\frac{1}{T_{m}}\right)}}{1+e^{\frac{-\Delta H_{D-N}}{R}\left(\frac{1}{T}-\frac{1}{T_{m}}\right)}}$$

where *FI*_340_ is the observed fluorescence emission at a given temperature, *T* is the temperature (in Kelvin), *α_N_* and *α_D_* are the intrinsic fluorescence of the native and denatured state, respectively, at 298 K, *β_N_* and *β_D_* are the slopes of the native and denatured state baselines, respectively, *R* is the gas constant and Δ*H_D-N_* is the enthalpy change of denaturation, which is related to the slope of the curve at the *T_m_* [48]. Since the thermal stabilization of *KcsA* by phospholipids occurs in a concentration-dependent manner, the following equilibria can be proposed:

(2) KcsA+L ↔KcsA∗L

Here, the stabilization of the native state of the protein relative to its denatured state as a consequence of the binding of a certain lipid, *L*, will depend on both the concentration of the lipid, [L], and the dissociation constant (*K_D_*) of the protein–ligand complex [49,50]. This dissociation constant can also be defined as:

(3) $$\Delta G_{unf}=\Delta G_{0}+R\;T\ln(1+\frac{\left[L\right]}{K_{D}})$$

where Δ*G*_0_ and Δ*G_unf_* are the free energy changes upon unfolding in the absence and presence of the ligand, respectively.

By combining all these equations together, the affinity of detergent-solubilized protein for different types of lipids can be calculated from the dependence of the *T_m_* parameter on the lipid concentration:

(4) $$\frac{\Delta T_{m}}{T_{m}}=\frac{T_{m}-{\left(T_{m}\right)}_{0}}{T_{m}}=\frac{R\;{\left(T_{m}\right)}_{0}}{\Delta H_{0}}\ln\left(1+\frac{\left[L\right]}{K_{D}}\right)$$

where *T_m_* and (*T_m_*)_0_ refer to the denaturation temperature (in kelvin) for the protein in the presence and absence of ligand, respectively, *R* is the gas constant, and Δ*H*_0_ is the enthalpy change upon protein denaturation in the absence of ligand.

Thus, fitting of experimental data to Equation (4) allows one to determine the equilibrium constant for the dissociation of the lipid-DDM-channel complex (K_D_) (Figure 3D). We should emphasize the importance of working with small amounts of lipids to calculate accurate K_D_ values, since there is a point where the solubilizing effect of the detergent becomes saturated and sample scattering becomes a complicating factor. Overall, these studies concluded that anionic phospholipids were much more efficient in increasing the protein’s thermal stability and binding KcsA with higher affinity than zwitterionic phospholipids (Figure 3B–D). Thus, the estimated dissociation constants (K_D_) for the single set of binding sites detected in the experiments shown in Figure 3 were 9 ± 6 µM and 0.5 ± 0.2 µM for dioleyl phosphatidylcholine (DOPC) and dioleyl phosphatidic acid (DOPA), respectively. Interestingly, even when there were no apparent differences in the effects of the zwitterionic phosphatidylcholine (PC) and phosphatidylethanolamine (PE) species, in the cases of the anionic species PA and PG, the affinity for KcsA is significantly higher in the former. Such effect could be ascribed to the ability of PA to increase its negative charge (to become dianionic) when in the presence of positively charged residues in its immediate surroundings [51]. It is also interesting to point out that similar stabilizing effects could be emulated by the addition of small amounts of alkyl sulfate derivatives, specifically those with 12–14 carbon atom long alkyl chains, suggesting that these latter anionic molecules can also bind to the non-annular lipid binding sites of KcsA and stabilize the protein against thermal unfolding. Regarding the effect of the acyl chain length, some functional studies have recently suggested that C18 phospholipids are optimal for the channel to become more active [52], by offering a proper membrane thickness and, therefore, an optimal stability of the intracellular gate of KcsA.

## 4. Ion Binding to the KcsA Selectivity Filter and Modulation by Lipids

The structural characterization of ion–protein interactions in KcsA was first described by crystallography and X-ray diffraction experiments [4,5,6,21]. Later studies used several other biophysical techniques, including electronic paramagnetic resonance (EPR), isothermal titration calorimetry (ITC), nuclear magnetic resonance (NMR) and, more recently, two-dimensional infrared (2D-IR) spectroscopy and time-resolved anisotropy with homo-FRET (Förster resonance energy transfer) analysis. These structural techniques are usually performed under equilibrium conditions, unlike the electrophysiological recordings used to measure functional properties (permeability, selectivity and inactivation processes), that are clearly far from the equilibrium point. Nevertheless, the affinity of KcsA for permeant and blocking cations determined by changes in structural parameters correlates reasonably well with functional properties of the channel (see below).

The simplest approach to characterize ion–protein interactions is to use the detergent-solubilized protein channel, in order to avoid the concomitant effect of the lipid bilayer. In KcsA, this micelle system was used in X-ray crystallography, ITC [17,20,53,54], NMR [55,56,57], and fluorescence techniques [19,22,58,59,60,61,62]. As indicated before, fluorescence approaches are always preferred in terms of protein concentration and sensitivity. Furthermore, working with diluted, less “crowded” samples, avoids the formation of supramolecular structures and their possible interference with the results (see next section). The thermal denaturation assay described in the previous section has been extensively applied to characterize cation binding to detergent-solubilized KcsA at low micromolar protein concentrations. In a first report, the heat-induced denaturation of the protein was used to characterize the binding of K^+^ and Na^+^ to the closed state (pH 7) of KcsA. Both cations greatly stabilize the channel against thermal denaturation [59], suggesting that synergistic effects of the metal-mediated intersubunit interactions are a major contributor to the stability of the tetrameric protein. The increase on the Tm shows two consecutive binding events for K^+^, and only one for Na^+^, the former cation being much more efficient in stabilizing the channel than the latter. These two consecutive binding events of different affinities found for K^+^ were ascribed to the non-conductive and conductive conformations of the selectivity filter previously described by crystallography and X-ray diffraction methods [4,5] (Figure 1B). That behavior was later confirmed for other permeant (Rb^+^, Tl^+^ and Cs^+^) and blocking (Ba^2+^) cations [22] and lend further support to the tenet that channel gating may be partially governed by concentration-dependent transitions between different affinity states of the selectivity filter. Moreover, the differences in affinities between permeant and non-permeant cations and the similarities in binding behavior within each of these two groups, correlate fully with their permeabilities relative to K^+^, suggesting that binding is an important determinant of the channel’s ion selectivity [22]. In the particular case of Na^+^, we also observed binding to KcsA with a K_D_ similar to that estimated electrophysiologically from channel blockade when working under non-competitive conditions (i.e., in the absence of K^+^) [59], but such affinity decreased drastically when the SF is pre-formed in the conductive conformation (i.e., presence of K^+^ in the mM range) [62], therefore contributing to the maintenance of channel selectivity.

As already explained in the introduction section, when the pH drops from neutral to acidic, the inner gate of KcsA opens, leading shortly afterwards to an allosteric conformational change of the SF from the conductive to the inactivated state in the presence of K^+^. The ion–protein interaction in the inactivated state was also studied by thermal denaturation, determining the affinity of different inactivated protein models to bind several permeant and nonpermeant cations [19]. The results show that the stack of ion binding sites in the inactivated filter models remains accessible to cations as they are in the resting channel state. Nonetheless, quantitative differences in the apparent K_D_’s of the binding processes reveal that the affinity for the binding of permeant cations to the inactivated channel models, mainly K^+^, decreases considerably with respect to the resting channel, which is also seen by solution NMR spectroscopy [57]. This is likely to cause a loss of K^+^ from the inactivated filter and consequently, to promote nonconductive conformations characteristic of the inactivated channel. Conversely, the complexity observed in permeation features (i.e., low open probability for K^+^ and very diminished inactivation in the presence of Rb^+^ or Cs^+^) cannot be explained just in terms of binding at equilibrium and likely relates to reported differences in the occupancy of the S2 and S3 sites by the permeant cations and/or kinetics contributions. In addition, some recent experiments performed by 2D-IR spectroscopy found that the occupation of the S1 site diminishes upon channel opening [63].

In an attempt to correlate these thermal stabilization data to conformational changes on the channel under similar experimental conditions (low protein concentration, detergent-solubilized system, room temperature, absence of antibody segments or external probes attached to the protein), we took advantage of the blue-shift of the intrinsic emission fluorescence spectra of the WT channel when Na^+^ is replaced for K^+^ in the buffer [58]. Modification of the shape and position of the spectra was quantified through the calculation of the center of mass <λ>. Monitoring of the variation of <λ> according to the K^+^ concentration in the presence of different amounts of Na^+^ revealed similar mM affinities to those observed through the thermal denaturation assay, suggesting that they are truly thermodynamic constants, independent of the technique used for their determination.

Since the intrinsic fluorescence of WT KcsA corresponds to the weighted average of the emission from five different Trp residues per subunit (Figure 3A), different channel mutants were developed in an attempt to dissect which regions of the channel were more responsive to ion binding. A first conclusion was that the triple mutant W26,87,113F had a very similar behavior to the WT protein in terms of the spectral changes caused by different types and concentrations of cations in the media. These observations suggest that W67 and W68, which are the only tryptophans present in the triple mutant, are most “sensitive” to the conformational changes associated with ion binding. Interestingly, these residues are located in the pore-helix, in close contact with the residues that form the selectivity filter, and therefore report on the conformation of this domain [59]. More recently, we went further and investigated the SF dynamics of a single Trp mutant of the potassium channel KcsA (W67) using polarized time-resolved fluorescence measurements. We found that in the closed state (pH 7), the W67–W67 intersubunit distances become indeed shorter as the average ion occupancy of the SF increases according to cation type and concentration, therefore complementing the information from X-ray crystallography, in which the protein conformational dynamics is usually compromised by the experimental conditions used, such as low temperature and the presence of an antibody segment attached to the extracellular loop. Moreover, we were able to monitor the transition between the collapsed and conductive conformational states by performing both steady-state and time-resolved anisotropy measurements as a function of either K^+^ or Na^+^ concentration. Both the steady-state fluorescence anisotropy and the calculated inter-subunit W67–W67 lateral distances obtained from analyzing the corresponding time-resolved data with the derived homo-FRET formalism varied pronouncedly in a dose-response manner with either the K^+^ or Na^+^ concentration used, resembling the results obtained by the thermal denaturation assay and confirming the association between cation binding and structural changes produced in the structures near the SF. In fact, whereas the first binding event of K^+^ (µM affinity) is associated with a modest thermal stabilization (~5–6 °C) and a subtle decrease in the intersubunit distances (~1 Å), the second K^+^ binding event (mM affinity) correlates with a much higher increase in the T_m_ (~35 °C) and further narrows down the average inter-tryptophan neighboring distances between the four subunits of W67 KcsA by ~3 Å.

A more complex characterization of the interaction of KcsA with relevant cations can be performed using lipid-reconstituted channels as the experimental system. In this case, ion–protein interactions could be modulated by the conformational changes associated with the channel incorporation into a membrane bilayer [17,64,65] and/or by the specific effect of certain types of lipids (mainly anionic phospholipids) bound to the intersubunit non-annular binding sites [19,30,31,34,39]. The most frequently used biophysical technique to characterize these interactions is solid state NMR. Several studies were performed, usually detecting changes in the chemical shifts of the ^74^TTVG^78^Y residues, combined with those of residues F103, I100 or G116, associated with closed/open states of the inner gate [66,67,68]. Even though the K_D_ values derived from those experiments do not always coincide with those from the detergent-solubilized channel, an interesting convergence is found in terms of the inactivated state of KcsA binding the K^+^ cation with a weaker affinity than the resting one. [69]. So far, no systematic structural studies have been published on the influence of the bilayer composition on KcsA–cation interactions and its consequences on the modulation of the open probability and C-type inactivation processes. Thus, the determination of the molecular basis of the modulation of ion conduction and C-type inactivation by the lipid composition of the bilayer, along with its link to the allosteric coupling between the inner and the outer gates to give rise to the different conformational channel states, is still pending. Simpler, but likely more limited, experimental systems should perhaps be used in this endeavor. For instance, the so called nanodiscs could substitute the more complex reconstituted vesicles and be used in fluorescence or solution NMR studies [70,71,72]. In addition, our group is using the mixed micelle system as an alternative, so that the combination of cation titration and the addition of small amounts of different types of lipids in the mixed micelles, should allow us to use the thermal denaturation assay to characterize the modulation of ion binding to the channel by the presence of lipids at the annular/non-annular binding sites. This mixed-micelle approach is still at a preliminary stage in our laboratory. However, experimental information is accumulating fairly rapidly and it is likely that these data would be available soon.

## 5. Lipid Modulation of Protein Assembly Processes in KcsA

### 5.1. Influence of Lipids on Folding and Tetramerization of KcsA

Folding and assembly of oligomeric membrane proteins has been in the spotlight for several decades. In the particular case of ion channels, the studies performed using KcsA as a model revealed some interesting features on these processes. Early studies performed in *E. coli* proved that the proton-motive force is essential for an efficient oligomerization of the channel [73] and most notable, that the presence of lipid vesicles, i.e., a phospholipid bilayer, is a minimal requirement to induce the tetramerization of KcsA when the channel is synthesized in a prokaryotic in vitro transcription-translation system [74]. KcsA can also be semisynthesized in vitro and then folded to the tetrameric state by its incorporation into lipid vesicles [38]. More recent studies confirmed that the channel expressed in cell-free systems aggregates in the absence of liposomes but spontaneously integrates into a liposome membrane, so that these lipid vesicles act similarly to chaperones, assisting in the formation of the channel-native structure [75,76]. It is interesting to point out that the composition of the lipid bilayer is also a key factor for an optimal protein insertion into the membrane. In the case of KcsA, vesicles with a large amount of PE and 20–30% of anionic phospholipids, such as phosphatidylglycerol (PG), presented the optimal ratio for an efficient membrane association [74,77].

KcsA is highly resistant to most conventional protein denaturants (SDS, urea or guanidine hydrochloride). Nevertheless, KcsA unfolding and dissociation into subunits can be attained in the presence of increasing concentrations of trifluoroethanol (TFE). Such effects include two successive cooperative transitions. First, at lower TFE concentrations, the tetrameric KcsA partly loses its secondary structure and dissociates into its constituent subunits. Under these conditions, simple dilution of the TFE permits a fairly efficient, but incomplete refolding and tetramerization of the protein in the detergent solution. The second cooperative transition occurring at higher TFE concentrations, however, leads to the irreversible denaturation of the protein [78].

Interestingly, the former folded tetramer to unfolded monomer transition becomes completely reversible when KcsA is solubilized in lipid-containing mixed micelles instead of just plain detergent micelles [33]. Moreover, reconstitution into asolectin giant liposomes of the refolded protein shows potassium channel activity, suggesting that channel function can also be regained upon refolding. The more effective lipid combination found corresponded to a mixture of dioleyl phosphatidylethanolamine (DOPE) and dioleyl phosphatidylglycerol (DOPG), the latter being an anionic phospholipid. In fact, when the TFE unfolding is carried out with liposome-reconstituted channels, the higher increment of the lateral pressure in the acyl chain region exerted by PE, compared to PC or PG, has been found to increase the KcsA resistance to TFE dissociation [79]. On the other hand, unfolding studies performed with hexafluoroisopropanol (HFIP) found that anionic phospholipids, particularly phosphatidic acid (PA), stabilized the KcsA tetramer against chemical denaturation by HFIP, and the effect was completely abolished by the deletion of the N-terminal domain [80]. Therefore, these results strongly suggest that lipids may also act as effectors in the tetramerization of KcsA.

### 5.2. Effects of Lipids on the Formation of KcsA Clusters and Their Gating Properties

**Figure 4:**
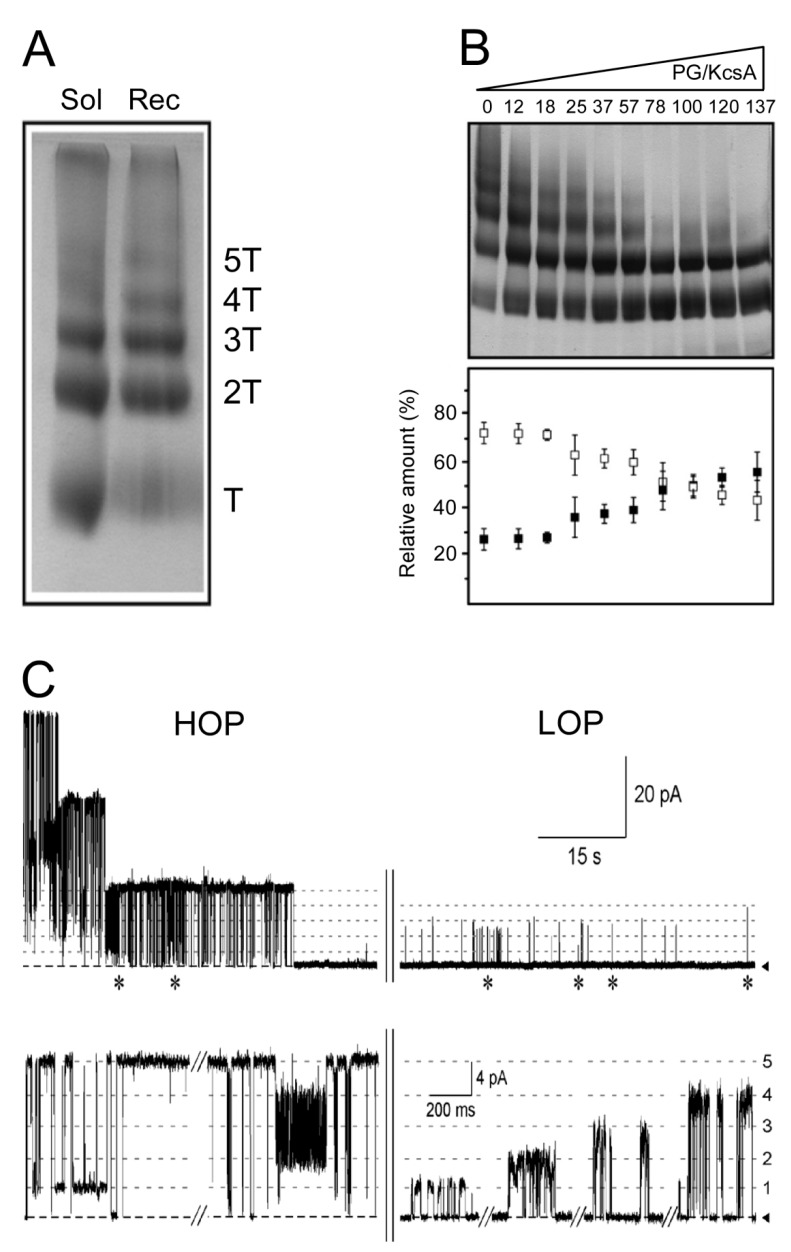
Lipids modulate clustering and gating activity in KcsA. (**A**) BN-PAGE of purified KcsA, either solubilized in DDM (Sol) or reconstituted in asolectin vesicle (Rec). T stands for KcsA tetramer. (**B**) BN-PAGE analysis of the effects of an anionic phospholipid (PG) on the disassembly of KcsA clusters in mixed micelles containing KcsA and PG at the indicated molar ratios. Clustered and individual KcsA are indicated by white and black squares, respectively. (**C**) High opening probability (HOP) versus low opening probability (LOP) activity patterns at +150 mV. Patch-clamp recordings of excised, inside-out membrane patches of purified KcsA reconstituted into giant liposomes made from asolectin phospholipids. The regions marked with asterisks in the two top recordings of (**C**), correspond to the recordings enlargements shown below. The numbers on the right correspond to the number of open channels.

Tetrameric KcsA has a tendency to self-associate above certain protein concentrations into supramolecular assemblies or clusters of different numbers of KcsA channels, both in vitro [81,82,83,84] and *in vivo*, suggesting that clusters may be even a native form of the channel in the bacterial membranes [85,86]. KcsA clusters can be easily detected by Blue Native-PAGE (BN-PAGE), a non-denaturing electrophoretic method [84] (Figure 4A). In addition to the individual KcsA tetrameric channel, other bands corresponding to two, three, four and five tetrameric channels have been detected. The larger KcsA clusters are probably organized from individual KcsA as well as from dimers of channels as the repeating building blocks [85]. A similar observation has been reported in the VDAC channel, where dimers of the channel appear to be the building block for the larger clusters [87]. Interestingly, KcsA clusters are detergent-labile since the larger clusters disassemble reversibly into smaller ones upon exposure to SDS-like alkyl sulfates [84]. These cluster-dissociating detergents have been shown to bind to the non-annular lipid binding sites on the protein (see above), thus, suggesting that such non-annular sites also mediate channel–channel interactions leading to cluster assembly. This is further supported by the observation that the occupation of the non-annular sites in KcsA by anionic phospholipids above a certain concentration, causes a progressive disappearance of the larger clusters in the BN-PAGE (Figure 4B), which has also been confirmed by other techniques [88].

In direct relation to the main scope of this review, the effects of anionic phospholipids on the assembly/disassembly of channel clusters seem to correlate with the appearance of different patterns of channel activity. Two main patterns of activity have been detected by patch-clamp recordings of excised, inside-out membrane patches of purified KcsA reconstituted into giant liposomes made from asolectin phospholipids: (i) a low opening probability (LOP) pattern in which channel openings are scarce and result primarily from single channel events or from occasional coupled gating of a few channels, and (ii) a high opening probability (HOP) pattern in which the channels are opened most of the time and exhibit positive cooperativity through coupled gating of a large number of channels (Figure 4C). The details on the ion conduction features in HOP and LOP patterns from KcsA reconstituted in the asolectin liposomes have been given previously [81]. The appearance of either one of these different opening probability patterns is dependent on the conditions and protein concentrations used for reconstitution of the channel protein, which reconciles the apparent discrepancies on the different levels of conductance of KcsA reported by other groups [89,90,91,92]. In essence, experiments in which the concentration of anionic phospholipids in the reconstituted liposomes is changed by adding these phospholipids into the reconstitution media suggest that the more complex HOP recordings indeed arise from large channel clusters, which are favored at low concentration of anionic lipids, whereas LOP recordings originate mostly from non-clustered, phospholipid-bound KcsA channels [88].

In an attempt to find a cause and effect relationship for the above phenomena, we used docking techniques partly based on X-ray crystallographic data, to model the consequences of the interaction of either an anionic phospholipid or another tetrameric channel at the non-annular site of KcsA [89]. These docking models were obtained years before the molecular dynamics simulations studies commented on in a previous paragraph [42], although essentially similar conclusions were derived from either technique. Basically, opposite to the channel–channel interaction, phospholipid binding to the channel non-annular site allows W67 to form the W67-E71-D80 inactivation triad, leaving the phospholipid-bound channel prone to inactivation. Obviously, a readily inactivating channel should produce recordings characterized by a low channel opening probability, that is, a LOP pattern of activity, as is indeed found experimentally. On the contrary, the inactivation triad in clustered KcsA is disrupted because channel–channel interaction causes W67 to swing away from its E71 and D80 partners. Therefore, these clustered channels would not be expected to inactivate as readily, as it is indeed the case in the observed HOP patterns of channel activity. Additional experiments using KcsA mutants with a deficient binding of anionic phospholipids to their non-annular sites, such as those related to the R64 and R89 positions, show the expected behavior in terms of both, the gating patterns and the disassemble of clusters; thus, strengthening the idea that indeed, competing lipid–channel and channel–channel interactions at non-annular lipid binding sites determine channel clustering and gating patterns.

Experimental data from other ion channels of excitable membranes, such as those in the heart and the nervous system, including the mammalian brain, also exhibit cooperative properties modulated by lipids, which are suggestive of clustering processes similar to that in KcsA [93,94,95,96,97,98,99,100,101,102,103]. This is to conclude that lipid-dependent processes such as those found in the KcsA model could be present in many other membrane proteins, including different ion channels.

## 6. Concluding Remark

By using KcsA as a model of K^+^ channels, this review illustrates how these membrane proteins integrate different input signals that are finally transduced into a conformational change of its selectivity filter, thus modulating the channel activity. Remarkably, binding of anionic lipids to the non-annular sites on the channel protein is a common event to most of these signaling paths and thus, these molecules can be considered as truly allosteric modulators of KcsA. Binding of anionic phospholipids influences the proper folding of the protein, increases its stability, alters the protein–protein interactions leading to channel clustering and strongly modulates the ion conduction properties. The characterization of the modulation of such activity by lipid molecules is an important task to find new pharmacological targets in order to develop drugs against channelopathies involving lipid-modulated channels.
